# Differences and Similarities in the Contributions of Phonological Awareness, Orthographic Knowledge and Semantic Competence to Reading Fluency in Chinese School-Age Children With and Without Hearing Loss

**DOI:** 10.3389/fpsyg.2021.649375

**Published:** 2021-04-12

**Authors:** Linjun Zhang, Tian Hong, Yu Li, Jiuju Wang, Yang Zhang, Hua Shu

**Affiliations:** ^1^Beijing Advanced Innovation Center for Language Resources and College of Advanced Chinese Training, Beijing Language and Culture University, Beijing, China; ^2^State Key Laboratory of Cognitive Neuroscience and Learning and IDG/McGovern Institute for Brain Research, Beijing Normal University, Beijing, China; ^3^Department of Applied Psychology, Beijing Normal University-Hong Kong Baptist University United International College, Guangdong, China; ^4^Peking University Sixth Hospital (Institute of Mental Health), Beijing, China; ^5^NHC Key Laboratory of Mental Health (Peking University) and National Clinical Research Center for Mental Disorders (Peking University Sixth Hospital), Beijing, China; ^6^Department of Speech-Language-Hearing Sciences and Center for Neurobehavioral Development, University of Minnesota, Minneapolis, MN, United States

**Keywords:** reading fluency, children with hearing loss, phonological awareness, orthographic knowledge, semantic competence 4

## Abstract

Compared with the large number of studies on reading of children with hearing loss (HL) in alphabetic languages, there are only a very limited number of studies on reading of Chinese-speaking children with HL. It remains unclear how phonological, orthographic, and semantic skills contribute to reading fluency of Chinese school-age children with HL. The present study explored this issue by examining the performances of children with HL on reading fluency and three linguistic skills compared with matched controls with normal hearing (NH). Specifically, twenty-eight children with HL and 28 chronological-age-matched children with NH were tested on word/sentence reading fluency (WRF/SRF), phonological awareness (PA) which was composed of onset/vowel/lexical tone awareness, orthographic knowledge (OK), and semantic competence (SC) which comprised animal word identification, pseudo-homophone detection, and word segmentation. Results showed that children with HL lagged behind their peers with NH in WRF/SRF and most of the phonological, orthographic, and semantic subskills except onset awareness and pseudo-homophone detection. Furthermore, the significant contributors to WRF differed between the two groups with PA being the significant contributor in the children with NH while OK being the significant contributor in the children with HL. However, the significant contributor to SRF did not differ between the two groups with SC being the only significant contributor. These results revealed not only between-group differences but also similarities in the relative contributions of PA, OK, and SC to reading fluency at both word and sentence levels, which has practical implications for developing better training programs to improve reading for children with HL.

## Introduction

Reading is one of the crucial skills for children to achieve academic success because much of what children need to learn is acquired through print once they reach roughly grade 4 (Chall, 1983). Research has consistently shown that the reading performance of children with hearing loss (HL) remains significantly worse than that of their hearing peers (Wauters et al., 2006; Spencer and Marschark, 2010; Cupples et al., 2014), which can hamper their success in various academic domains (Ingvalson et al., 2020). Nowadays more and more children with HL are fitted with appropriate amplification, such as hearing aid and/or cochlear implant (CI), which provides them with access to speech sounds to some extent and thus bring substantial gains in their spoken language and reading ability (Tomblin et al., 2005; Vermeulen et al., 2007; Spencer and Oleson, 2008; Niparko et al., 2010). However, children with HL except for those with early cochlear implantation continue to lag behind their peers with normal hearing (NH) on measures of reading ability although much effort has been put into reading training and literacy education for them (Geers, 2003; Johnson and Goswami, 2010; Geers and Hayes, 2011; Qi and Mitchell, 2012; Harris et al., 2017).

There are large variations in reading outcomes among the children with HL, and some children's reading ability is even above the average grade level of their normal-hearing peers (Geers, 2003; Vermeulen et al., 2007; Geers et al., 2008). Many previous studies intended to improve reading instructions for children with HL have identified factors that contribute to reading achievements, among which phonological awareness (PA), orthographic knowledge (OK), and semantic competence (SC) are the main linguistic skills (Dillon et al., 2012; Von Muenster and Baker, 2014; Chan and Yang, 2018; Wass et al., 2019; Ingvalson et al., 2020).

### Contribution of Phonological Awareness (PA) to Reading in Children With NH and HL

As a subset of phonological processing skills, PA refers to the ability to identify and manipulate speech sounds (Hatcher et al., 1994) and has been known to play an important role in reading development of children with NH irrespective of whether the writing system is alphabetic or non-alphabetic (McBride-Chang and Ho, 2000; McBride-Chang and Kail, 2002; Oakhill and Cain, 2012; Rakhlin et al., 2014). In an alphabetic writing system such as English, the association between PA and reading is underpinned by the mapping of graphemes onto phonemes during reading. Specifically, if there is difficulty in identifying a sequence of phonemes in speech, it is inaccessible to represent spoken words as a sequence of corresponding graphemes during reading (Cupples and Iacono, 2000). In a meta-analysis of 235 studies which examined the association between PA and word reading, Melby-Lervåg et al. (2012) found that inferior word reading was significantly related with poorer PA. Previous studies that investigated the cognitive dynamics underlying reading fluency also revealed substantial contribution of PA to word (Landerl et al., 2019) and text reading fluency (Georgiou et al., 2008). In a non-alphabetic writing system such as Chinese, the strong links between PA and character recognition (Ho and Bryant, 1997; Li et al., 2012; Liu et al., 2017) and sentence reading fluency (Lei et al., 2011; Xue et al., 2013; Bai et al., 2020) have also been confirmed although characteristics of Chinese scripts make the grapheme-phoneme mapping different from that of an alphabetic writing system.

The association between PA and reading in children with prelingual HL is hotly debated. Some researchers argue that children with HL must learn to represent spoken phonology because the same processes are involved in reading for children with HL as children with NH (Musselman, 2000; Perfetti and Sandak, 2000). For example, Colin et al. (2007) found that sensitivity to phonemic structure is significantly related to word reading accuracy for French-speaking children with CIs and those with NH. Dillon et al. (2012) found that the correlation between PA and sentence reading accuracy was still significant after controlling for children's age and speech perception skills in English-speaking children with CIs aged 6–14 years. However, some studies reported no significant association between PA and literacy in children with HL. For example, Izzo (2002) found that phonemic awareness was not significantly correlated with reading ability which was measured by a story retelling task in children with HL of primary school age. Results of two longitudinal studies showed that PA was not a significant predictor to word reading accuracy in the early stage of reading development among children with HL (Kyle and Harris, 2010, 2011).

A very limited number of studies on reading of children with HL in the non-alphabetic Chinese writing system also revealed inconsistent results. For example, Ching and Nunes (2015) reported significant contribution of PA (lexical tone awareness) to word recognition accuracy in Chinese-speaking children with HL aged 7–12 years, whereas Chan and Yang (2018) found that PA (onset awareness and rime awareness) did not make a unique contribution to the amount of recognized Chinese characters in children with HL of grade 2. In a recent study by Zhao et al. (2019), unique contribution of phonological awareness (onset awareness) to Chinese sentence reading fluency was found for children with HL of grades 5–6. Taken together, evidence on the relationship between PA and reading in children with HL is equivocal due to a lack of consistent findings across studies, which is in sharp contrast to the well documented association between PA and reading in children with NH. This inconsistency could stem at least partly from the differences in the reading measures (e.g., word vs. sentence/text reading, reading accuracy vs. fluency) and a wide range of demographic variables that might affect PA and/or reading outcomes in children with HL (e.g., chronological age, age at CI implantation, cognitive ability). Further investigation is needed to determine how these variables affect the associations between PA and reading in children with HL, especially children of non-alphabetic languages such as Chinese.

### Contribution of Orthographic Knowledge (OK) to Reading in Children With NH and HL

In addition to PA, OK which refers to the ability to use visual-orthographic information during processing printed words (Barker et al., 1992; Rakhlin et al., 2014) is closely related to reading in alphabetic and non-alphabetic writing systems (O'Brien et al., 2011; Elhassan et al., 2017; Bai et al., 2020). In an alphabetic writing system, learning to read begins with learning the alphabetic principle by associating letters with their names and sounds (Shmidman and Ehri, 2010). As reading skills develop, a direct lexical route is possible for highly practiced words that can be read by visual processing of the letters without phonological mediation (Coltheart, 1978; Frith, 1985). Previous studies have shown that OK is significantly associated with reading fluency in alphabetic writing systems. For example, O'Brien et al. (2011) found that OK contributed to passage reading fluency but not spelling performance in English-speaking children of grades 1–3, and Papadopoulos et al. (2016) found that OK was a significant predictor to reading fluency among Greek-speaking children of grades 1–2. In a recent study, Rakhlin et al. (2019) found that orthographic processing skill was the strongest predictor to reading fluency in two groups of Russian-speaking children with good and poor reading ability. Given the visual-orthographic complexity of Chinese characters, it has been long proposed that orthographic processing skills are more closely associated with Chinese reading than alphabetic reading (Siok and Fletcher, 2001; Chung et al., 2008). For example, Tan et al. (2005) found that writing skills, through which children learned to decompose Chinese characters into stroke patterns and reassemble these patterns into a square unit, were strongly associated with character reading fluency. Li et al. (2012) found that OK contributed significantly to Chinese character recognition in primary school children, and Liu et al. (2017) further confirmed that OK had a strong effect on Chinese character recognition accuracy and word reading fluency in children across 1–6 grades.

Considering the reduced or deprived sensitivity to speech sounds in children with HL, it is likely that they may be able to read through visually based processes by direct mappings between a grapheme and combination of graphemes (printed words) and their signed or spoken counterparts (Goldin-Meadow and Mayberry, 2001; Allen et al., 2009). For example, Geers (2003) and James et al. (2009) found that children with late cochlear implantation relied more on orthographic information than controls with NH during phonological processing. Domínguez et al. (2014) found that adults with profound HL performed lexical decision task based on orthography better than their counterparts with NH. Such a superiority in orthographic processing was evident even in primary school children with HL (Domínguez et al., 2019). These results indicate that readers with HL may store representations of graphemes and printed words in a special way, in which the orthographic information is able to be processed with little phonological support. As the Chinese writing system has an extremely opaque sound-form mapping system with complex visual-orthographic characteristics, Chinese-speaking children with HL may resort to OK more than alphabetic language children in order to aid reading. To our best knowledge, only one study has explored the contribution of OK to Chinese reading in children with HL. Specifically, Ching and Nunes (2015) found that one measurement of Chinese orthographic knowledge (semantic radical awareness) contributed significantly to two-character word recognition in children with HL aged 7–12 years. However, reading ability was measured only through word recognition accuracy in this study. How OK contributes to Chinese reading fluency at both word and sentence levels in children with HL needs further investigation.

### Contribution of Semantic Competence (SC) to Reading in Children With NH and HL

TIn addition to the code-based skills such as PA and OK underlying reading, meaning-based skills (i.e., semantic competence) such as receptive vocabulary, quality of word representations, morphological awareness and listening comprehension are necessary to understand the decoded words, sentences and texts. For example, a number of studies found that large proportions of reading comprehension could be explained by variance in vocabulary once children with NH passed the early stages of reading development (Protopapas et al., 2007; Olson et al., 2011; Wass et al., 2019). Further, knowledge of word meaning contributed directly to reading comprehension (Ouellette and Beers, 2010; Perfetti and Stafura, 2014). Because of the correspondence between syllables, characters and morphemes in Chinese, morphological awareness has been found to be a strong predictor to a wide range of reading measures of children with NH, including Chinese character recognition, reading accuracy and fluency at both word and sentence levels (Shu et al., 2006; Chen et al., 2009; Liu et al., 2017).

TIn children with HL, meaning-based skills have also been found to play important roles in reading (Lederberg et al., 2014; Wass et al., 2019). For example, in alphabetic writing systems, Connor and Zwolan (2004) found that vocabulary growth had a positive effect on passage comprehension in primary and middle school children with CIs and Von Muenster and Baker (2014) reported strong correlations between receptive/expressive language skills and passage reading comprehension in children with CIs aged 5–13 years. In non-alphabetic writing system, Ching and Nunes (2015) and Chan and Yang (2018) found that morphological awareness significantly predicted Chinese word recognition in primary school children with HL. Listening comprehension and receptive vocabulary knowledge also contributed significantly to early development of reading comprehension (Chan and Yang, 2018).

To summarize, the linguistic factors of phonological, orthographic and semantic skills, which are associated with reading ability of children with NH, might also contribute to reading achievements of children with HL. Two outstanding issues have emerged from the review of previous literature. First, the findings regarding the association between PA and reading in children with HL is inconsistent with each other. It is also unclear whether PA contributes to reading ability of Chinese children with HL for their non-alphabetic writing system. Second, although OK might be more closely associated with Chinese reading than with alphabetic reading, it has attracted little attention among the limited number of studies on reading of Chinese-speaking children with HL.

The purpose of this study was to compare the performances of children with HL and NL on word/sentence reading fluency (WRF/SRF) and a series of phonological, orthographic, and semantic subskills. Reading is a complex process involving the construction of literal and inferred meanings from print, among which word reading fluency (WRF) and sentence reading fluency (SRF) are two essential components (Klauda and Guthrie, 2008). While WRF mainly recruits cognitive operations at lower-levels such as visual feature extraction, phonological and orthographic decoding, SRF is associated with higher-level processes such as syntactic analysis and semantic integration in addition to decoding skill. Previous studies have also shown that there are developmental changes in how orthographic decoding influences semantic and phonological processing and how WRF and SRF may make differential contributions to reading comprehension with the existence of substantial individual differences (Jenkins et al., 2003; Eberhard-Moscicka et al., 2015; Kim, 2015). In the present study, we were more interested in examining the contributions of PA, OK, and SC to Chinese reading fluency at both word and sentence levels in children with HL compared to children with NH in order to determine whether the same prediction models of WRF/SRF would apply to both groups of children. We hypothesized that (1) children with HL would have poorer performance than children with NH on WRF/SRF and all the phonological and semantic subskills, but have equal or even better performance on orthographic processing, and (2) one or more of the three linguistic skills, i.e., PA, OK, and SC could make independent contributions to WRF/SRF with PA and OK most likely contributing to WRF and SC contributing to SRF beyond demographic factors and general intelligence in children with NH and HL. Beyond these two specific predictions, the search remained exploratory in nature for the similarities and differences between the two groups in the relative contributions of the three linguistic skills to reading.

## Methods

### Participants

Twenty-eight children with HL (18 boys and 10 girls) were recruited from rehabilitation centers and special education schools in Beijing, and 28 chronological age-matched children with NH (19 boys and 9 girls) were recruited from mainstream schools in Beijing. All the participants with HL were prelingually deafened and late cochlear implanted (implantation age > 2 years) with 10 of them implanted in the right ear and the other 18 in the left ear. They were all profoundly deaf (unaided pure-tone threshold average ≥ 90 dB HL) and were users of Cochlear's Nucleus 22 or 24 devices (Cochlear Beijing Limited, Beijing, China) with 17 of them used hearing aids prior to cochlear implantation. They all enrolled in oral educational programs and used oral language as the main communication mode. The speech production accuracy of each child with HL was assessed by a pilot Chinese character naming study in which the child was asked to read aloud 40 familiar Chinese characters. Pronunciations of the Chinese characters included all the 21 initials, 39 rimes, and 4 tones. Results of the pilot study showed that each child with HL could pronounce all the Chinese segmental and suprasegmental phonemes although some of the phonemes were pronounced with systematic variations (e.g., the pitch height of Tone 4 is a bit lower than normal). None of the children with HL or NH had a medical history of neurological, psychiatric, or neuropsychological disorders. The current study was approved by the Institutional Review Board (IRB) of the State Key Laboratory of Cognitive Neuroscience and Learning at Beijing Normal University. A written informed consent was obtained from the participants or their legal guardians.

### Measures

#### General Intelligence

Children's general intelligence was measured with the Chinese version of Raven's standard progressive matrices (Zhang and Wang, 1985) in order to control for any potential influence of general cognitive ability on reading and the linguistic skills. It is a standardized test which includes 60 items in total with five sets of 12 items each. Each item that is presented in printed format consists of a target matrix with a missing part. Children were required to choose the best figure from six to eight choices to complete the target matrix. One point was given for a correct answer.

#### Word and Sentence Reading Fluency

Reading fluency was assessed at both word and sentence levels, which were measured with the one-minute and three-minute reading tasks, respectively. The two tasks have been widely used in previous studies on reading ability of Chinese-speaking children with HL and NH and showed adequate reliability and validity (e.g., Liu et al., 2017; Zhao et al., 2019; Bai et al., 2020). In the one-minute reading task, children were required to read aloud as quickly as possible 180 two-character Chinese words (e.g., “我们”, “we”) that were randomly arranged in 20 rows of 9 each. The score was tallied on the number of words read correctly. The first author who was an expert in phonetics and familiar with the speech of children with NH and HL monitored the children's performances. Children with NH pronounced all the words without any errors, and children with HL were also judged to pronounce all the words correctly by reference to their pronunciations in the pilot study. In the 3-min reading task, children were asked to read silently as quickly as possible 100 simple sentences that gradually increased in length and judge whether the statement was true or false (e.g., “蚂蚁比大象小很多”, “An ant is much smaller than an elephant”). The score was the summed number of characters in correctly judged sentences. All the characters, words and meanings of sentences in the two tests were familiar to the children with HL and NH based on our pilot study.

#### Phonological Awareness

Three PA subtests were conducted to measure three subskills of phonological awareness, i.e., onset, vowel and lexical tone awareness, respectively (Liu et al., 2017; Xia et al., 2018). Specifically, children were given a number of characters with high frequency arranged in three 11 × 14 matrices and were instructed to cross out the characters whose pronunciation started with the onset “b” (onset awareness subtest, e.g., “八”pronounced as “ba1”), contained the vowel “u” (vowel awareness subtest, e.g., “土” pronounced as “tu3”) or the dipping tone (lexical tone awareness subtest, e.g., “扫” pronounced as “sao3”) within 80 s, respectively. Examples and practice trials were provided before each formal subtest to ensure that all children understood the tasks. The score of each subtest was the number of correctly identified characters minus the number of falsely chosen per minute, and the three scores were then combined to form a composite score of PA (Xia et al., 2018).

#### Orthographic Knowledge

A non-character recognition test was conducted to measure OK (Zou et al., 2012; Liu et al., 2017; Xia et al., 2018). During this test, children were shown a number of items with characters and non-characters randomly arranged in an 11 × 14 matrix and were asked to cross out the non-characters from the real characters within 80 s. All of the non-characters had ill-formed structures, i.e., they contained real components which were placed in the illegal positions (e.g., “
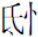
”). Examples and practice trials were provided before the timed testing to ensure that all children understood the instructions. The score was the number of correctly identified non-characters minus the number of falsely chosen per minute.

#### Semantic Competence

Three subtests, specifically animal word identification, pseudo-homophone detection and word segmentation were conducted to measure semantic competence (Liu et al., 2017; Xia et al., 2018). During the animal word identification subtest, animal and non-animal words that were randomly arranged in an 11 × 10 matrix were presented and children were asked to cross out all the animal words within 35 s. During the pseudo-homophone detection subtest, children were presented with real words and pseudo-homophone words randomly and were instructed to cross out the pseudo-homophone words within 45 s. Both real words and pseudo-homophone words consisted of two characters and the pseudo-homophone words were formed by replacing one character with a homophone (e.g., the pseudo-homophone word “平果” was formed by replacing the first character of the real word “苹果”, “apple”). During the word segmentation subtest, children were presented with a continuous line of printed words without inter-word spaces and were required to identify the word boundaries with slashes (e.g., “苍蝇/熊/香蕉”, “fly/bear/banana”) within 60 s. Examples and practice trials were provided in each subtest. The score of each subtest was the number of correct responses minus the number of false responses per minute, and the three scores were then combined to form a composite score of semantic competence (Xia et al., 2018).

### Data Analysis

We adopted three major statistical approaches to analyze the data. Independent-sample *t*-tests were first used to assess any group difference in all the measures. Correlation analyses were then carried out to examine the relationship among chronological age/age of cochlear implantation, general intelligence, the two reading fluency skills and the three linguistic skills. Finally, hierarchical regression analyses were undertaken to examine the relative contributions of PA, OK, and SC to WRF and SRF in children with NH and HL.

## Results

Performance scores of children with NH and HL on all the measures are summarized in Table 1. Independent-samples *t*-tests revealed that children with HL performed significantly poorer than their peers with NH on all the measures except for onset awareness and pseudo-homophone detection.

**Table 1:** Descriptive statistics and group comparisons on all the measures (mean ± SD) for the children with HL and NH.

	**Children with HL**	**Children with NH**	***t* (54)**	***p value***
Chronological age (years)	15.71 (3.83)	17.01 (4.33)	−1.19	0.24
Implantation age (years)	6.93 (2.91)	-	-	-
General intelligence	25.07 (4.64)	28.79 (2.85)	−3.61	0.001
Word reading fluency	79.00 (22.37)	116.95 (18.95)	−6.85	<0.001
Sentence reading fluency	254.10 (170.39)	457.01 (100.00)	−5.35	<0.001
Phonological awareness				
Onset awareness	24.29 (9.59)	25.40 (6.46)	−0.51	0.614
Vowel awareness	17.55 (6.30)	24.14 (7.43)	−3.50	0.001
Lexical tone awareness	6.05 (6.12)	18.44 (5.96)	−7.28	<0.001
Orthographic knowledge				
Orthographic recognition	55.82 (14.05)	68.12 (19.10)	−2.75	0.008
Semantic competence				
Animal word identification	33.24 (10.37)	44.75 (9.22)	−4.39	<0.001
Pseudo-homophone detection	21.27 (9.70)	25.83 (7.92)	−1.93	0.059
Word segmentation	16.52 (10.61)	29.01 (7.35)	−5.12	<0.001

*HL, hearing loss. NH, normal hearing*.

The correlations among chronological age/implantation age, general intelligence, WRF, SRF, PA, OK, and SC in the two groups are summarized in Table 2. The results showed that in children with HL, WRF was significantly correlated with OK and SC, but not with PA, general intelligence, chronological age, and age of cochlear implantation, while SRF was significantly correlated with OK, SC, chronological age, and age of cochlear implantation, but not with PA and general intelligence. By contrast, in children with NH, WRF was significantly correlated with PA, general intelligence and chronological age, but not with OK and SC, while sentence reading fluency was significantly correlated with SC, general intelligence and chronological age, but not with PA and OK.

**Table 2:** Bivariate correlations among age, general intelligence, predictors, word reading fluency and sentence reading fluency separately children with HL and NL.

**Measure**	**1**	**2**	**3**	**4**	**5**	**6**	**7**	**8**
Children with HL (*n* = 28)								
1. Word reading fluency	–							
2. Sentence reading fluency	0.36	–						
3. General intelligence	0.25	0.18	–					
4. Phonological awareness	0.28	0.37	0.19	–				
5. Orthographic knowledge	0.71^**^	0.58^**^	0.18	0.56^**^	–			
6. Semantic competence	0.40^*^	0.86^**^	0.26	0.51^**^	0.75^**^	–		
7. Chronological age	0.34	0.45^*^	−0.04	0.65^**^	0.56^**^	0.56^**^	–	
8. Implantation age	0.37	0.54^**^	0.09	0.48^*^	0.49^**^	0.56^**^	0.60^**^	–
Children with NH (*n* = 28)								
1. Word reading fluency	–							
2. Sentence reading fluency	0.54^**^	–						
3. General intelligence	0.60^**^	0.44^*^	–					
4. Phonological awareness	0.67^**^	0.31	0.47^*^	–				
5. Orthographic knowledge	0.18	0.22	0.51^**^	0.29	–			
6. Semantic competence	0.26	0.64^**^	0.49^**^	0.09	0.53^**^	–		
7. Chronological age	0.49^**^	0.56^**^	0.43^*^	0.36	0.42^*^	0.67^**^	–	

*HL, hearing loss. NH, normal hearing*.

* *p < 0.05*,

** *p < 0.01*.

To further examine the contributions of the three linguistic skills, i.e., PA, OK, and SC, to WRF and SRF in the two groups of children, we carried out four fixed-order hierarchical multiple regressions with chronological age and age of cochlear implantation being entered into the models as steps 1, general intelligence as step 2, and the three linguistic skills as step 3 (Tables 3, 4). The results showed that OK was the only significant predictor to WRF in children with HL, but PA and general intelligence were significant predictors to WRF in children with NH. However, SC was the only significant predictor for SRF in both groups.

**Table 3:** Hierarchical multiple regressions predicting word reading fluency and sentence reading fluency for children with HL.

**Measures**	**Step**	**Predictor**	**β**	***t***	**R^**2**^**	**ΔR^**2**^**	**Adjusted R^**2**^**
Word reading fluency	1	Chronological age	0.18	0.78	0.16	0.16	0.09
		Implantation age	0.27	1.15			
	2	Chronological age	0.21	0.94	0.21	0.05	0.12
		Implantation age	0.22	0.97			
		General intelligence	0.24	1.29			
	3	Chronological age	0.07	0.34	0.62	0.41	0.51
		Implantation age	0.17	0.96			
		General intelligence	0.21	1.43			
		Phonological awareness	−0.23	−1.21			
		Orthographic knowledge	1.00	4.66^***^			
		Semantic competence	−0.43	−1.92			
Sentence reading fluency	1	Chronological age	0.19	0.90	0.32	0.32	0.26
		Implantation age	0.43	2.08^*^			
	2	Chronological age	0.21	1.00	0.34	0.02	0.26
		Implantation age	0.40	1.92			
		General intelligence	0.15	0.91			
	3	Chronological age	−0.06	−0.38	0.77	0.43	0.70
		Implantation age	0.14	0.99			
		General intelligence	−0.05	−0.45			
		Phonological awareness	−0.05	−0.37			
		Orthographic knowledge	−0.15	−0.88			
		Semantic competence	0.97	5.62^***^			

*HL, hearing loss*.

* *p < 0.05*,

*** *p < 0.001*.

**Table 4:** Hierarchical multiple regressions predicting word reading fluency and sentence reading fluency for children with NH.

**Measures**	**Step**	**Predictor**	**β**	***T***	**R^**2**^**	**ΔR^**2**^**	**Adjusted R^**2**^**
Word reading fluency	1	Chronological age	0.49	2.85^**^	0.24	0.24	0.21
	2	Chronological age	0.28	1.68	0.42	0.19	0.38
		General intelligence	0.48	2.83^**^			
	3	Chronological age	0.27	1.44	0.63	0.21	0.55
		General intelligence	0.42	2.37^*^			
		Phonological awareness	0.46	2.82^*^			
		Orthographic knowledge	−0.27	−1.67			
		Semantic competence	−0.02	−0.07			
Sentence reading fluency	1	Chronological age	0.56	3.24^**^	0.31	0.31	0.28
	2	Chronological age	0.46	2.40^*^	0.36	0.05	0.30
		General intelligence	0.24	1.26			
	3	Chronological age	0.21	0.88	0.49	0.13	0.35
		General intelligence	0.12	0.54			
		Phonological awareness	0.09	0.42			
		Orthographic knowledge	−0.20	−0.99			
		Semantic competence	0.52	2.12^*^			

*NH, normal hearing*.

* *p < 0.05*,

** *p < 0.01*.

## Discussion

This study investigated the performances of Chinese school-age children with HL on reading fluency and a series of linguistic subskills compared with their peers with NH, and the differences and similarities between the two groups in the contributions of phonological, orthographic and semantic skills to reading fluency. The detrimental effects that hearing impairment has on the development of reading ability and related subskills have been investigated by many previous studies in alphabetic (Geers, 2003; Johnson and Goswami, 2010; Geers and Hayes, 2011; Qi and Mitchell, 2012; Harris et al., 2017) and non-alphabetic languages (Ching and Nunes, 2015; Zhao et al., 2019). Results of the present study that children with HL performed worse than age-matched controls on reading fluency at both word and sentence levels and most of the phonological and semantic subskills, therefore, are consistent with those of the previous studies. The two groups, however, did not differ in their performances on one phonological (onset awareness) and one semantic (pseudo-homophone detection) subskills, indicating that children with HL have comparable abilities to decode onset during orthography-phoneme conversion and to access orthographic vocabulary during lexical decision. Interestingly, in contrast to our prediction, children with HL showed poorer performance on OK than their peers with NH. Our result was different from previous findings that children with HL showed equivalent and even better performance on orthographic processing compared with their peers with NH (Vermeulen et al., 2007; Domínguez et al., 2014, 2019; Ching and Nunes, 2015). The inconsistency might be related with different tasks adopted to measure OK in these studies (e.g., visual word recognition, meta-phonological awareness and radical awareness). In the current study, OK was measured through a character decision task. Our result that all the children finished the task without any mistake indicate that children with NL and HL have full knowledge of orthographic structure of Chinese characters used in the test. More importantly, the poorer performance of children with HL indicate that hearing impairment does not lead to compensatory improvement of efficiency in visual processing of the orthographic structure of Chinese characters. It needs to be pointed out that Chinese writing system is characteristic of complex visual-orthographic properties. Orthographic processing skills, therefore, can be measured at different levels. For example, illegal Chinese characters can be designed in different types: line drawings with no conventional stroke patterns, pseudo-characters with well-formed structures and components, non-characters with well-formed structures and ill-formed components, and non-characters with ill-formed structures and real components (Li et al., 2012; Xue et al., 2013). The current study included only real characters and non-characters with ill-formed structures and real components. Further investigations are needed to clarify the effects of hearing loss on accuracy and efficiency of orthographic processing by using other tasks and experimental items.

The contribution of PA to Chinese reading in children with NH at single-character and two-character word levels has been widely acknowledged although Chinese writing system is non-alphabetic (McBride-Chang and Ho, 2000; McBride-Chang and Kail, 2002; Shu et al., 2008; Li et al., 2012; Xue et al., 2013). Our result that PA contributed significantly to WRF of children with NH, therefore, is consistent with the previous findings. However, the result that OK did not make a significant contribution to WRF of children with NH seems to contradict some of the previous findings which showed that OK played an important role in Chinese reading (Ho et al., 2003; Li et al., 2012; Xue et al., 2013; Ching and Nunes, 2015). This discrepancy might be caused by the differences in tasks, experimental items and especially measurements (accuracy vs. reaction speed) adopted in the present and previous studies (also refer to the last paragraph of the discussion section) and needs further investigation. By contrast, OK was the only significant contributor to WRF in children with HL although the results of zero-order correlations revealed that both OK and SC were significantly correlated with WRF. The difference in the relative contributions of PA and OK to WRF in children with NH and HL revealed that children with HL relied more on OK than their peers with NH, indicating a strategic shift of decoding bias or dependence toward visual orthographic processing due to hearing impairment.

In addition to coding-based skills (e.g., PA and OK) which are the primary processes recruited during Chinese reading at the levels of single-character and two-character word, meaning-based skills play important roles in reading at sentence and text levels (Xue et al., 2013; Ching and Nunes, 2015; Liu et al., 2017; Zhao et al., 2019). In the current study, SC was a composite score of three subtests, namely animal word identification, word segmentation and pseudo-homophone detection. The three semantic tasks measured various aspects of semantic ability with the former two mainly reflecting vocabulary knowledge and the latter being strongly associated with morphological awareness (Liu et al., 2017; Xia et al., 2018). Our result that SC contributed to SRF in both groups of children indicate that children with HL and NH rely more on SC than PA and OK during sentence reading, reflecting similarity in the contributions of linguistic skills to reading at sentence level.

Although the relationship between general intelligence and reading ability is not the focus of the present study, it is noteworthy that general intelligence made an independent significant contribution to WRF but not SRF in children with NH while it did not contribute independently to WRF or SRF in children with HL. In previous studies on reading abilities of children with NH and HL, general intelligence was often considered as a controlled variable together with other variables such as chronological age and CI age for their potential influences on reading. Findings on the relationship between general intelligence and reading ability have been mixed. For example, Ching and Nunes (2015) found nonsignificant contribution of general intelligence to word recognition accuracy in Chinese-speaking children with HL and NH aged 7–12 years. By contrast, Zhao et al. (2019) reported significant contribution of general intelligence to SRF in younger children with NH (3–4 grades) and older children with HL (5–6 grades). Such significant contribution, however, was absent in older children with NH and younger children with HL (Zhao et al., 2019). Thus, further research is needed to determine whether general intelligence contributes independently to the development of reading skills in Chinese-speaking children with or without hearing loss.

Taken together, findings of the present study have some important implications for a better understanding of the similarities and differences in the developmental trajectory of reading-related linguistic subskills and their contributions to reading in children with HL and NL. First, although children with HL lagged behind their peers with NH in most of the orthographic, phonological, and semantic skills, they exhibited comparable performance on onset awareness and pseudo-homophone detection, indicating that children with HL are able to develop some appropriate phonological and semantic skills despite their overall disadvantages in linguistic skills. Second, the significant contributors to reading fluency at word level differed between the two groups, while the significant contributor to reading fluency at sentence level kept the same in the two groups, indicating that different and similar strategies were respectively, adopted by children with NH and HL during reading depending on whether syntactic processing and contextual semantic integration are involved. Accordingly, our findings have several pedagogical implications for children with HL. First, reading training program can make full use of the intact visual ability to enhance orthographic skills in order to improve WRF. Second, semantic skills should be more emphasized as sentence reading is at a higher stage of reading development than word reading (Chall, 1983). Third, intervention programs aimed to improve phonological awareness should focus on vowel and lexical tone awareness rather than onset awareness.

The present study is limited in several aspects. First, the sample size is not large enough to divide the children into subgroups, e.g., elementary-school and middle- school ages, while previous studies have revealed that the relative contributions of phonological, visual-orthographic and morphological skills to WRF and SRF in Chinese-speaking children with HL and NH could be modulated by developmental stages (Li et al., 2012; Xue et al., 2013; Liu et al., 2017; Zhao et al., 2019). Second, only three reading-related subskills were measured, while there might exit some other linguistic skills that play important roles in reading development. For example, syntactic process is necessary for reading at sentence and text levels, but we did not measure syntactic competence here. Furthermore, all the children were CI users with functional hearing. They had some degree of speech perception ability, which might also contribute to reading at both word and sentence levels. These issues need to be addressed in future studies with larger sample size and more reading-related subskills measured.

## Conclusion

The present study revealed differences and similarities between children with and without hearing loss in the contributions of phonological, orthographic, and semantic skills to word and sentence reading fluency. Our findings have practical implications for training program development aimed to improve reading and reading-related linguistic skills in children with HL.

## Data Availability Statement

The original contributions presented in the study are included in the article/supplementary material, further inquiries can be directed to the corresponding author/s.

## Ethics Statement

The studies involving human participants were reviewed and approved by the Institutional Review Board (IRB) of the State Key Laboratory of Cognitive Neuroscience and Learning at Beijing Normal University. Written informed consent to participate in this study was provided by the participants' legal guardian/next of kin.

## Author Contributions

LZ, TH, and JW: conceptualization, data collection, analysis, and manuscript preparation. YL, HS, and YZ: conceptualization and manuscript preparation. All authors contributed to the article and approved the submitted version.

## Conflict of Interest

The authors declare that the research was conducted in the absence of any commercial or financial relationships that could be construed as a potential conflict of interest.
